# PoseEdit: enhanced ligand binding mode communication by interactive 2D diagrams

**DOI:** 10.1007/s10822-023-00522-4

**Published:** 2023-07-29

**Authors:** Konrad Diedrich, Bennet Krause, Ole Berg, Matthias Rarey

**Affiliations:** 1 Universität Hamburg, ZBH—Center for Bioinformatics, 20146 Hamburg, Germany; 2 Present Address: Capgemini, 10785 Berlin, Germany

**Keywords:** Protein–ligand complexes, Molecular interactions, Mutable molecule visualization, 2D structure diagrams, Pose diagrams, PoseView, Protein Data Bank

## Abstract

In this article, we present PoseEdit, a new, interactive frontend of the popular pose visualization tool PoseView. PoseEdit automatically produces high-quality 2D diagrams of intermolecular interactions in 3D binding sites calculated from ligands in complex with protein, DNA, and RNA. The PoseView diagrams have been improved in several aspects, most notably in their interactivity. Thanks to the easy-to-use 2D editor of PoseEdit, the diagrams are extensively editable and extendible by the user, can be merged with other diagrams, and even be created from scratch. A large variety of graphical objects in the diagram can be moved, rotated, selected and highlighted, mirrored, removed, or even newly added. Furthermore, PoseEdit enables a synchronized 2D-3D view of macromolecule-ligand complexes simplifying the analysis of structural features and interactions. The representation of individual diagram objects regarding their visualized chemical properties, like stereochemistry, and general graphical styles, like the color of interactions, can additionally be edited. The primary objective of PoseEdit is to support scientists with an enhanced way to communicate ligand binding mode information through graphical 2D representations optimized with the scientist’s input in accordance with objective criteria and individual needs. PoseEdit is freely available on the Proteins*Plus* web server (https://proteins.plus).

**Supplementary Information:**

The online version contains supplementary material available at 10.1007/s10822-023-00522-4.

## Introduction

In the broad field of life sciences, the analysis of ligand interactions in biomacromolecular binding sites is crucial. For instance, medicinal chemists are required to visually investigate the activity of their candidate compounds obtained by molecular docking or virtual screening during a drug design endeavor. Also, they might want to concisely present the activity of their final compounds to others in reports, presentations, or scientific publications. The use of graphical representations is a common medium for communicating such information to scientists. Despite the lack of geometrical details, two-dimensional (2D) depictions of macromolecule-ligand complexes and the corresponding interactions are widely used in scientific research and commonly preferably chosen over three-dimensional (3D) counterparts. The exploration of a binding site via a 3D viewer is usually more time-consuming, and the proper usage requires some practice. Furthermore, the amount of buried visual information in a 2D screenshot could prevent a concise overall picture of how and with what a ligand interacts. The dimensional simplification of a binding site towards a planar arrangement of its constituents limits the content of spatial information. Still, it brings the interactions of the ligand and the interaction partners into the scientist’s focus. This type of visualization renders these critical aspects clearly visible and facilitates an instant overview that is not feasible in any 3D presentation.

To the best of our knowledge, there are only a few published and freely accessible tools that automatically generate 2D diagrams of ligand interactions from 3D input structures: LigPlot + [1, 2], LeView [3], and PoseView [4–6]. Furthermore, some commercial modeling and screening software packages like MOE [7] and the Python library ProLIF [8] contain related functionalities. All mentioned tools are desktop applications except PoseView, which is accessible via a web server in addition. It should also be noted that these tools are aged, given that they were released more than ten years ago. The low number, limited accessibility, and high age of the existing 2D ligand interaction visualizer indicate a potential for further development. Considering the high and continuously growing number of citations of MOE, LigPlot + , and PoseView in particular, it is evident that there is a high demand for these tools and, therefore, also a potential interest in further tool development. In our opinion, especially the tool’s user interfaces would benefit from some improvements regarding design and functionality to better support key scientific tasks.

Various issues may become apparent while examining 2D macromolecule-ligand interaction diagrams, such as:

- Intersecting lines representing interactions
- Overlapping graphical objects like structural objects
- Interaction lines crossing other graphical objects like a text label
- Crowded diagrams due to numerous graphical objects located too close to each other
- Missing or unnecessary chemical information, such as an interaction with a specific residue or the protonation state of an atom
- Unattractive graphical styles in the diagram, like low aesthetic quality of the structural drawings, the font type of atom labels, etc.

Due to such deficiencies that might be objective, like in the case of graphical collisions, or more of subjective nature regarding, for instance, the chemical information content, the ligand’s layout, the overall arrangement of residues or graphical styles, a ligand binding mode may not be satisfactorily represented for users. Therefore, they might consider a diagram inadequate for investigating or presenting the ligand’s interactions.

**Fig. 1:**
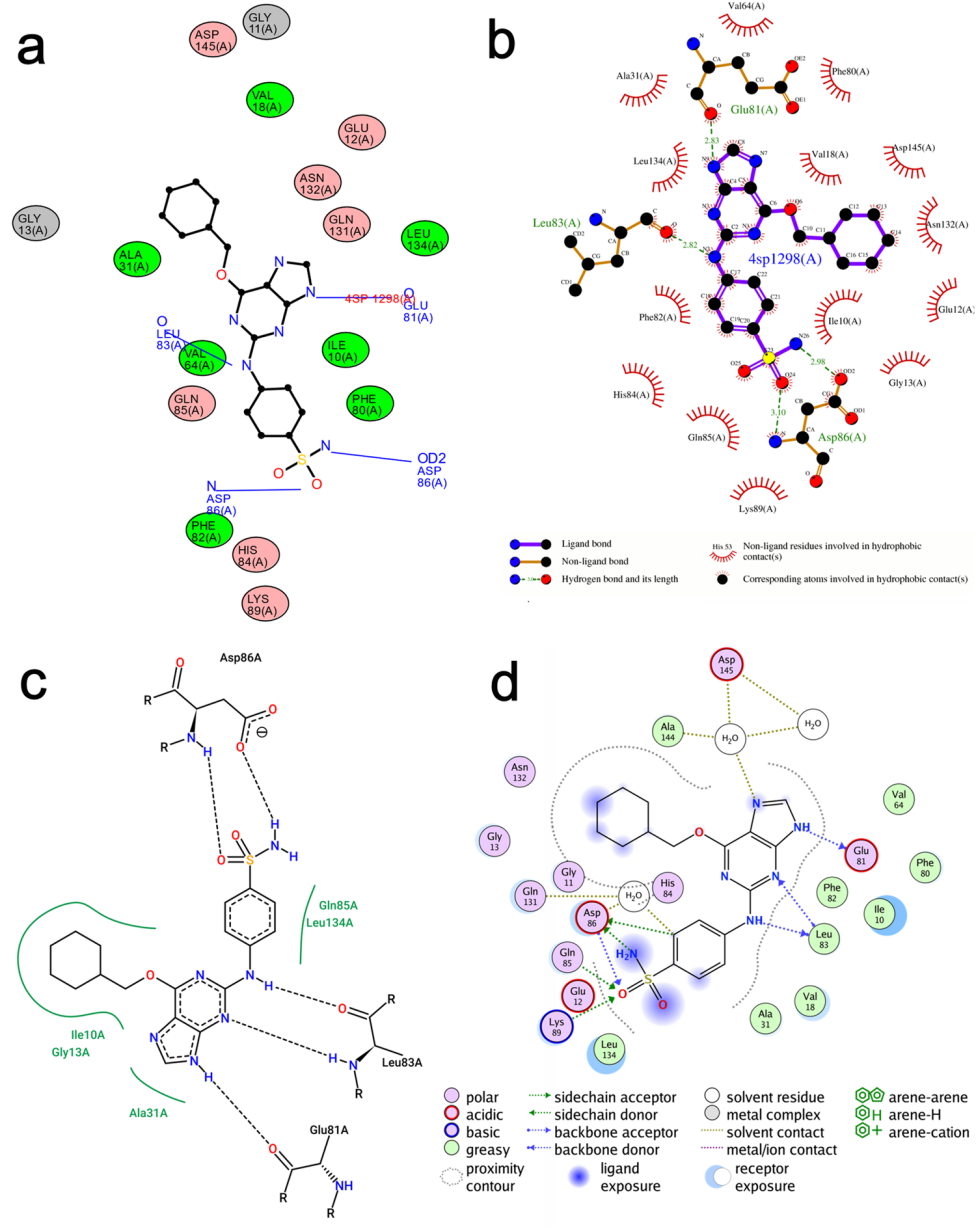
Diagrams of different tools of the inhibitor 4-[[6-(cyclohexylmethoxy)-7H-purin-2-yl]amino]benzenesulfonamide in complex with a cyclin-dependent kinase (PDB code: 1H1S). **a** LeView. **b** LigPlot + . **c** PoseView. **d** MOE

The diagrams produced by the above-mentioned tools vary significantly regarding the chemical information content, graphical styles, and occurrence of objective aesthetic deficiencies. Figure 1 shows the diagrams of LeView, LigPlot + , PoseView, and MOE of the inhibitor 4-[[6-(cyclohexylmethoxy)-7H-purin-2-yl]amino]benzenesulfonamide in complex with a cyclin-dependent kinase (PDB code: 1H1S) [9] and Table 1 illustrates the diagrams generated by these tools in comparison.

**Table 1:** Comparison of the diagrams created by LeView, LigPlot + , PoseView, and MOE

	PoseView	LeView	LigPlot +	MOE
Ligand representation	- Skeletal	- Skeletal	- Skeletal	- Skeletal
Interaction partner representation	- Skeletal	- Circle	- Skeletal	- Circle
Multiple ligands	−	−	+	−
IUPAC	+	−	−	−
Hydrogen bonds	+	+ (H_2_O-mediated)	+ (H_2_O-mediated)	+ (H_2_O-mediated)
Hydrophobic contacts	+	Near residues	+	Near residues
pi–pi	+	−	−	+
Cation-pi	+	−	−	+
pi-H	−	−	−	+
Ionic	−	−	−	+
Metal	+	−	−	+
Covalent bonds	−	−	+	+
Charges	+	−	−	+
Explicit hydrogens	+	−	−	+
Bond order assignment	Automated	CIF-based	CIF-based	Automated

Based on the differences of the diagrams, users can address the previously described issues to a limited extent by choosing the most suitable tool. Some examples are given in the following.

Users who want to display a ligand binding mode with a high level of chemical detail may consider PoseView or LigPlot + as an appropriate choice. Using PoseView, characteristics of the ligand binding mode, such as the interacting atoms, can be easily identified due to the drawing of structures in atomic detail. Moreover, structures are specified with further details like explicit polar hydrogen atoms and charge symbols. Only residues involved in hydrophobic contacts are represented by text labels that annotate splines placed around the ligand. Like PoseView, LigPlot + also draws complete structures except hydrophobically interacting residues in atomic detail but without showing further details like charges or polar hydrogen atoms. Residues with hydrophobic contacts to the ligand are drawn as labeled arcs with spikes extending in the ligand’s direction.

If users are more interested in an overall picture of the binding site or in a collision-free layout, MOE and LeView should be used. MOE and LeView reduce the level of detail by showing only the ligand in atomic detail and interacting structures as circles or text labels. This approach reduces collisions and consequently permits the visualization of not only the ligand’s interaction partners but also surrounding non-interacting structures. In MOE, all non-interacting residues, cofactors, and solvent molecules within a 4.5 Å cut-off radius of the ligand atoms are shown. In LeView, users can adjust the cut-off distance to include non-interacting residues and water molecules.

PoseView might be a convenient choice if the aesthetic quality of the chemical drawings is important to users. It is the only tool that strictly adheres to the Union of Pure and Applied Chemistry (IUPAC) [10] guidelines, which define a style of the depiction of chemical structures applied by the vast majority of scientists. The other tools tend to deviate from IUPAC ideals, resulting in issues like inconsistent bond lengths and angle sizes, or they draw structures as circles or text labels.

Users who want to visualize a wide variety of interaction types of a ligand binding mode may consider PoseView and MOE as the best options. Both tools include hydrophobic contacts, hydrogen bonds, pi-pi interactions, cation-pi interactions with protein and nucleic acid residues, and interactions with metals. LigPlot + considers fewer interaction types, including hydrophobic contacts and hydrogen bonds to protein and nucleic acid residues and metal interactions. LeView depicts hydrogen bonds only. In addition to direct hydrogen bonds, MOE, LigPlot + , and LeView can also display hydrogen bonds to residues that are mediated by water.

Despite the wide range of options offered by these tools, users may fail to find a viable workaround to the various issues previously described or be forced to an unsatisfactory compromise. For instance, users might want to reduce collision in a highly complex diagram by choosing MOE. Still, they want to keep certain favored aspects of other tools like the IUPAC-based depiction style of PoseView. In both cases, the users are forced to accept the diagram’s graphical styles, chemical information content, and objective aesthetic deficiencies.

The manual modification of a diagram after its generation is an approach that could help users to satisfy objective aesthetic requirements and subjective preferences about what is displayed and how. For example, the appropriate rearrangements of the diagram content could resolve intersections, overlaps, and overcrowded scenes. While the diagrams of PoseView are static, this approach is feasible to a varying extent in LeView, LigPlot+, and MOE, which provide interactive diagrams through a 2D editor interface. Table 2 presents a comparison across the editing features offered by these three tools.

**Table 2:** Comparison of the 2D editor interfaces of PoseView, LeView, LigPlot + , and MOE

	PoseView	LeView	LigPlot +	MOE
Diagram modification features				
Graphical styles (sizes, colors, etc.)	−	+	+	+
Interactive object types	−	− Structures	− Structures − Atoms − Text labels	− None
Object translation	−	+	+	−
Object rotation	−	−	+	−
Object removal	−	+	−	−
Mirror structure at bond	−	−	+	−
Merge multiple diagrams	−	−	+	−
Usability features				
Editing history	−	−	+ (undo of the last ten structural movements)	−
Diagram export	PDF, PNG, SVG	PNG, JPG, GIF, PDF, SVG, EPS, TXT	PS, DRW	PNG, JPG, EPS, PS, BMP, TIF, EMF + , SVG
Diagram import	−	−	DRW	−
3D visualization	−	−	+	+
Interactions list	−	+	+	+
Diagram legend	−	−	+	+
Diagram rotation	−	+ (45° intervals)	−	+
Diagram translation	−	−	+	−
Zoom in/out of diagram	−	−	+	−
Diagram reset	−	+	−	+
Diagram recentering	−	−	+	−

With the intent to create the most user-friendly and useful frontend possible for the manual post-processing of 2D ligand interaction diagrams, we compiled a list of features that specifically address the issues mentioned above, as well as the respective limitations of the existing tools. Based on that list, we extended PoseView, resulting in a new graphical frontend PoseEdit, which we present in this paper. In addition, we also aimed to address some graphical and informational shortcomings in the PoseView diagrams and consequently modified those in this regard. In the following, we will primarily focus on the newly built 2D editor of PoseEdit and its features. We will then showcase the usage of the 2D editor and discuss the benefits of its features for improving interactive 2D ligand interaction diagrams from a user’s perspective.

## Methods

### Features

The PoseView diagrams have been enhanced regarding graphical style and chemical information content, but most importantly, their interactivity. The key features of PoseEdit and its improvements over PoseView and the other tools are summarized in the following and include:

- A maximized accessibility through its implementation as a web application, which is freely accessible as part of the Proteins*Plus* [11–13] web server’s tool collection (https://proteins.plus)
- Interactive diagrams presented through a 2D editor with an intuitive interface design
- A large variety of interactive objects in the diagram, including all structures (the ligand, metal ions, protein and nucleic acid residues) and their atoms and bonds, hydrophobic contact splines and their spline control points, interactions, and text labels
- Extensive manual modification options through the translation, rotation, highlighting, hiding, mirroring, adding, removing, and editing of visualized chemical properties and graphical styles of interactive diagram objects
- The merging of multiple diagrams
- The export of the diagram and its legend in Scalable Vector Graphics (SVG) format
- Additional 2D editor features for a user-friendly overall diagram editing experience, such as the zooming, translation, rotation, and recentering of the diagram, the reset of the diagram to its initial unmodified state, the selection of multiple interactive diagram objects for editing them as a group, an editing history enabling undo/redo of all user changes and the export of diagrams in the JavaScript Object Notation (JSON) format that can be reimported for sharing and further editing
- An improved comprehension and exploration of the ligand binding mode through several interactive info sections of the editor and a simultaneously and synergistically inspectable 3D representation, which is synchronized with the 2D ligand interaction diagram
- An exportable JSON and Text Document (TXT) file that can be parsed for obtaining corresponding textual information
- An increased aesthetic quality of the PoseView diagram due to graphical style choices such as the depiction of bonds by using a color gradient, a minimal atom radius, within which bonds and interactions are not allowed to extend such that collisions are reduced, the drawing of interactions by colored lines with dashes of equal length and the visualization of amino acid side chains up to the Cα atom
- A more detailed description of the ligand binding mode by the depiction of covalent bonds of the ligand to residues and a new reparametrized interaction model based on the tools GeoMine [14–16] and Protoss [17, 18] that also annotates ionic interactions with residues and assigns pi-pi and cation-pi interactions to single aromatic rings rather than to entire aromatic ring systems

### Proteins*Plus* user interface and PoseEdit integration

**Fig. 2:**
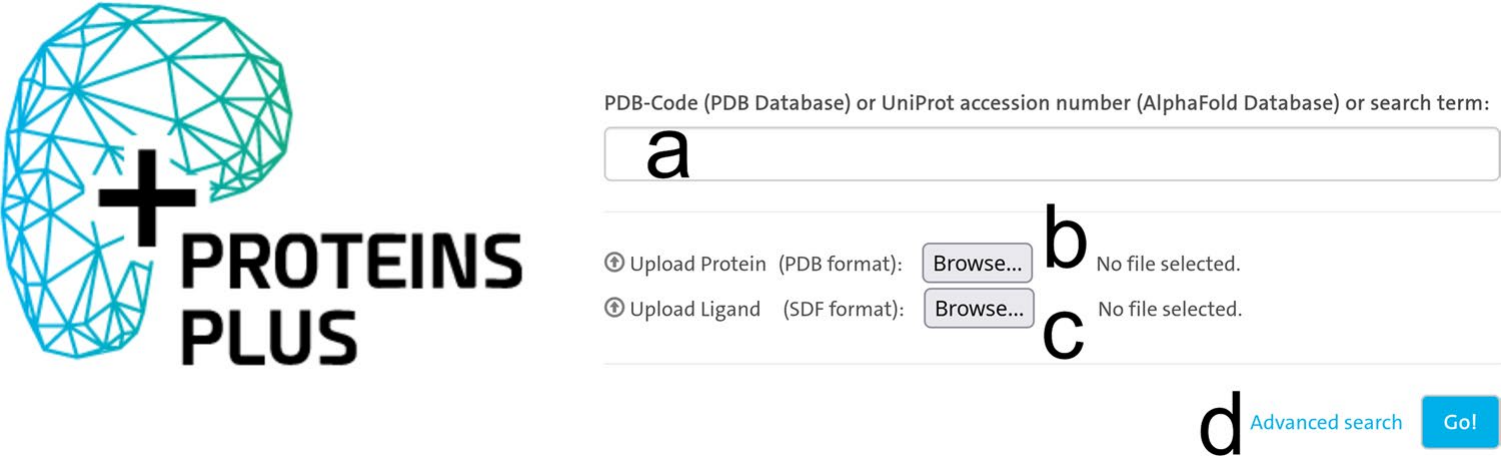
Input area of the landing page of the Proteins*Plus* web server. **a** Text field for the specification of an input structure via a Protein Data Bank identifier or UniProt accession number for the AlphaFold database. **b** Upload button for the upload of a structure file in PDB format. **c** Upload button for additional ligands in SDF format. **d** Link to the keyword search functionality

The input for PoseEdit is provided on the Proteins*Plus* landing page (Fig. 2) through the specification of a Protein Data Bank (PDB) [19] identifier, UniProt accession number for accessing a structure in the AlphaFold [20] database (Fig. 2a), or by the upload of a structure file in the PDB format (Fig. 2b). Additionally, users can upload ligands in the Structural Data File (SDF) format that are docked into a binding site of the input structure (Fig. 2c). If the users do not have a structure of interest yet, they can obtain a list of potential input candidates by querying the PDB or AlphaFold databases with keywords via the linked keyword search functionality (Fig. 2d). As an alternative to the Proteins*Plus* web site, PoseEdit can also be used in a more direct and automated way via the Representational State Transfer Application Programming Interface (REST API) of Proteins*Plus*, whose usage documentation can be found on the web page.

**Fig. 3:**
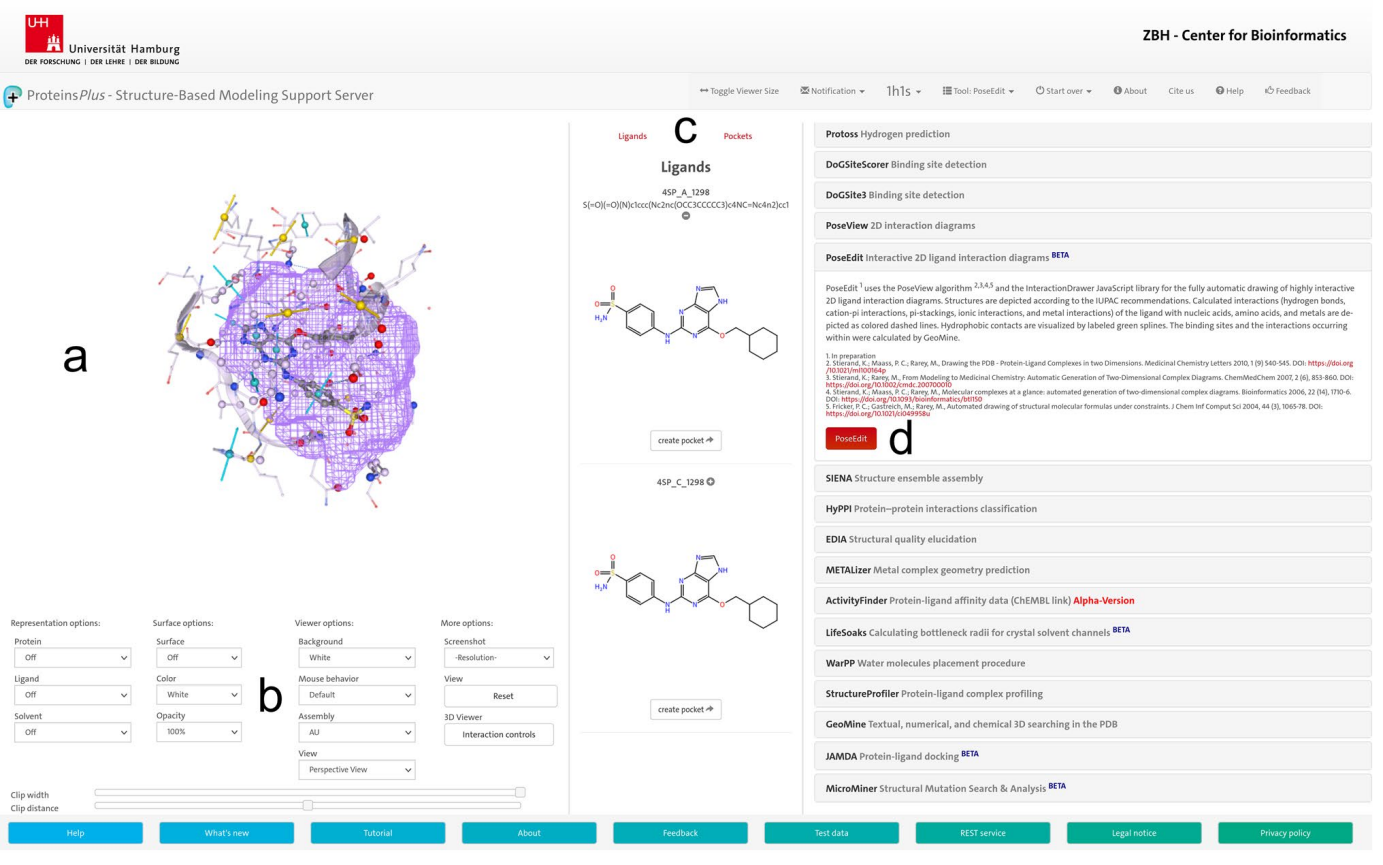
Main page of the Proteins*Plus* web server. **a** 3D viewer showing a 3D binding site of the inhibitor 4-[[6-(cyclohexylmethoxy)-7H-purin-2-yl]amino]benzenesulfonamide in complex with a cyclin-dependent kinase (PDB code: 1H1S). **b** 3D viewer control panel. **c** Togglable lists with ligands and on-the-fly calculated binding sites of the input structure. **d** list of tools, e.g., PoseEdit

After Protein*Plus* has preprocessed the input, users are forwarded to the main page (Fig. 3), which is divided into three primary sections: the 3D visualization section (Fig. 3a) on the left shows the input structure in a 3D viewer, which can be set up via a control panel below (Fig. 3b). The users can switch between two scrollable lists in the central section (Fig. 3c). The names, Simplified Molecular Input Line Entry System (SMILES) strings, and 2D diagrams of all ions and small molecules of the input structure such as solvent molecules, cofactors, and inhibitors are included in the *Ligand list*. The *Pocket list* contains empty and ligand-bound binding sites that are calculated on-the-fly from the input structure [21] and which can be separately visualized in the 3D viewer, along with further highlighted details such as the intermolecular interactions. In the tools section (Fig. 3d) on the right, users can select PoseEdit from the tool list, specify an input ligand from the *Ligand list* and start the diagram calculation. After the calculation is finished, the 2D editor of PoseEdit with the 2D ligand interaction diagram appears in the tools section. Furthermore, a link for the later retrieval of the calculated and unmodified diagram is provided.

### The PoseEdit editor

**Fig. 4:**
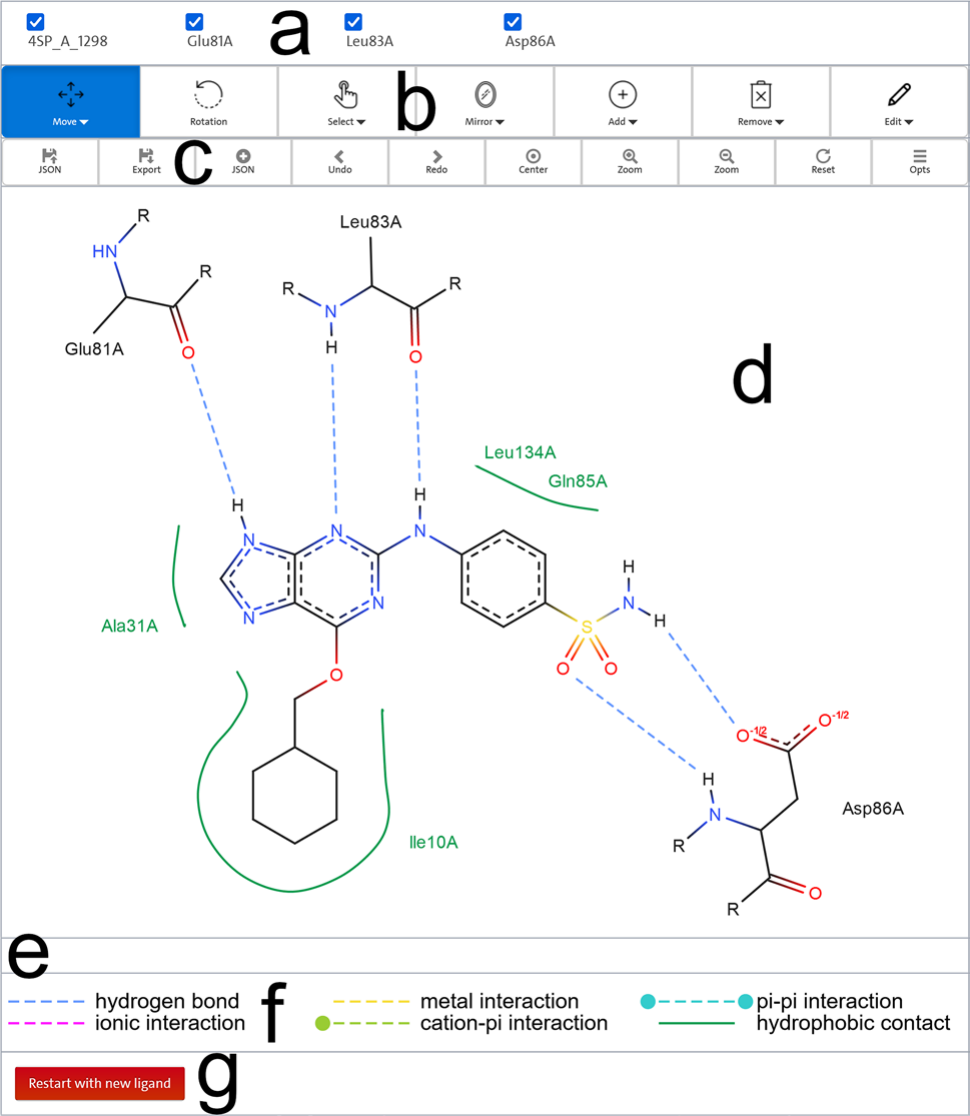
2D editor of PoseEdit showing a diagram of the inhibitor 4-[[6-(cyclohexylmethoxy)-7H-purin-2-yl]amino]benzenesulfonamide with the internal Proteins*Plus* ID 4SP_A_1298 interacting via hydrogen bonds and hydrophobic contacts with a cyclin-dependent kinase (PDB code: 1H1S). **a** Info section that contains the names of all structures in the diagram. **b** Buttons for the activation of a diagram editing mode. **c** Buttons for the handling of diagram files and additional editor controls. **d** Drawing area displaying the 2D ligand interaction diagram. **e** Info section that shows information about atoms, bonds, and structures hovered over with the mouse pointer in the diagram or 3D viewer. **f** Legend that illustrates the supported interaction types. **g** Restart button

The 2D editor of PoseEdit (Fig. 4) provides a top panel that consists of an info section with two toolbars below. The info section on the top (Fig. 4a) lists the names of all structures of the diagram. The two toolbars below contain buttons labeled with text and icons that indicate their functions. Users can select a diagram editing mode in the toolbar at the top (Fig. 4b). All modes are described in Table 3. A mode is activated by clicking its corresponding button, whose color then turns blue. Modes with an inverted triangle icon next to the text label of the corresponding button require further specification by the users for activation. When users click on such a mode button, a drop-down list appears allowing users to choose a mode-specific option. For example, users can define whether the Move mode affects single atoms, bonds, rings, or the complete structure. The activated mode can be applied by performing the required left mouse click and click-and-drag operations in the drawing area. The second toolbar (Fig. 4c) below contains buttons for downloading and uploading a diagram in different file formats and buttons that execute actions that directly modify the drawing area, such as the reset of the diagram to its initial unmodified state. Below the top panel is the drawing area (Fig. 4d) that shows the calculated 2D ligand interaction diagram and two additional info sections. The first one (Fig. 4e) displays information about atoms, bonds, and structures that are hovered over with the mouse pointer in the diagram or the 3D viewer. The second section (Fig. 4f) contains a legend that illustrates the supported intermolecular interaction types and their corresponding colors. A new PoseEdit calculation can be performed with different ligands from the *Ligand list* by clicking the restart button below (Fig. 4g). By moving the mouse pointer over any control element of the 2D editor, such as the button of a diagram editing mode, a tooltip with corresponding usage information appears.

**Table 3:** Description of PoseEdit’s diagram editing modes

mode	options	function
Move	Structure freedom level	Move the scene, a structure, structure circle, hydrophobic contact spline and its control points, or annotation. When a structure or structure circle is moved, all linked hydrophobic contact splines and annotations are also moved. When *Structure freedom level* is set to *Atoms and bonds* or *Rings*, the mode affects not the complete structure but its atoms and bonds or rings, respectively
Rotation	–	Rotate the scene, a structure, structure circle, or hydrophobic contact spline around their midpoints. When a structure or structure circle is rotated, all linked hydrophobic contact splines and annotations are also rotated
Select	Click Lasso Rectangle	Select objects in the drawing area by mouse click or with a rectangular or lasso selection tool. Deselect an object by clicking on it again and deselect everything by clicking in the blank of the drawing area. Selected objects are highlighted, which is synchronized with the 3D viewer and visible in the downloadable SVG. Selected atoms, bonds, structures, and structure circles can be moved, rotated, and removed together
Mirror	Bond Line	Mirror a structure at a bond or a structure or hydrophobic contact spline at a user-defined line that goes through its midpoint. When a structure is mirrored, all linked hydrophobic contact splines and annotations are also mirrored
Add	Annotation Atom with covalent bond Atom–atom interaction Cation-pi interaction Pi–pi interaction Explicit H with covalent bond Hydrophobic contact Structure	Specify an object type and add a new object of this type to the diagram. For an atom, annotation, or structure, several properties can be specified via a form
Remove	Structure freedom level	Remove a structure, structure circle, hydrophobic contact spline or its control points, annotation, or interaction. When a structure or structure circle is removed, all linked interactions, hydrophobic contact splines, and annotations are also removed. When *Structure freedom level* is set to *Atoms and bonds* or *Rings*, the mode affects not the complete structure but its atoms and bonds or rings, respectively
Edit	Annotation Atom Bond Structure	Specify an object type and edit the properties of a diagram object of this type via a form

### Technical implementation details

The frontend was developed with HTML, Vanilla JavaScript, and the Bootstrap 3 library (https://getbootstrap.com). The 2D diagrams are implemented by Scalable Vector Graphics. SVGs are created and rendered interactive by the InteractionDrawer JavaScript library, which is based on D3.js (https://d3js.org). The InteractionDrawer library was newly developed for that purpose, and its code is available on GitHub (https://github.com/rareylab/InteractionDrawer). The SMILES parsing, required for adding new structures specified by SMILES, is achieved by integrating the SmilesDrawer [22] JavaScript library. The 3D viewer is implemented with the NGL library [23, 24]. The Ruby on Rails framework (https://rubyonrails.org) was used to develop the backend of the webserver.

## Application

Our showcase of PoseEdit’s features is based on the structure of a lysine-specific histone demethylase 1A (LSD1, PDB code: 5LGT) in complex with the inhibitor 4-methyl-*N*-[2-[[4-(1-methylpiperidin-4-yl)oxyphenoxy]methyl]phenyl]thieno[3,2-b]pyrrole-5-carboxamide (Proteins*Plus* identifier: 6W3_A_902) and an flavin adenine dinucleotide (FAD) cofactor (Proteins*Plus* identifier: FAD_A_901) in the same binding site [25]. First, we will demonstrate how users can verify the chemical information content of a diagram. Next, we will show how users can optimize a diagram according to objective layout quality issues. Last, we will exemplify how users can further customize a diagram by editing its chemical information content and graphical restyling.

### Verification of the chemical information content

**Fig. 5:**
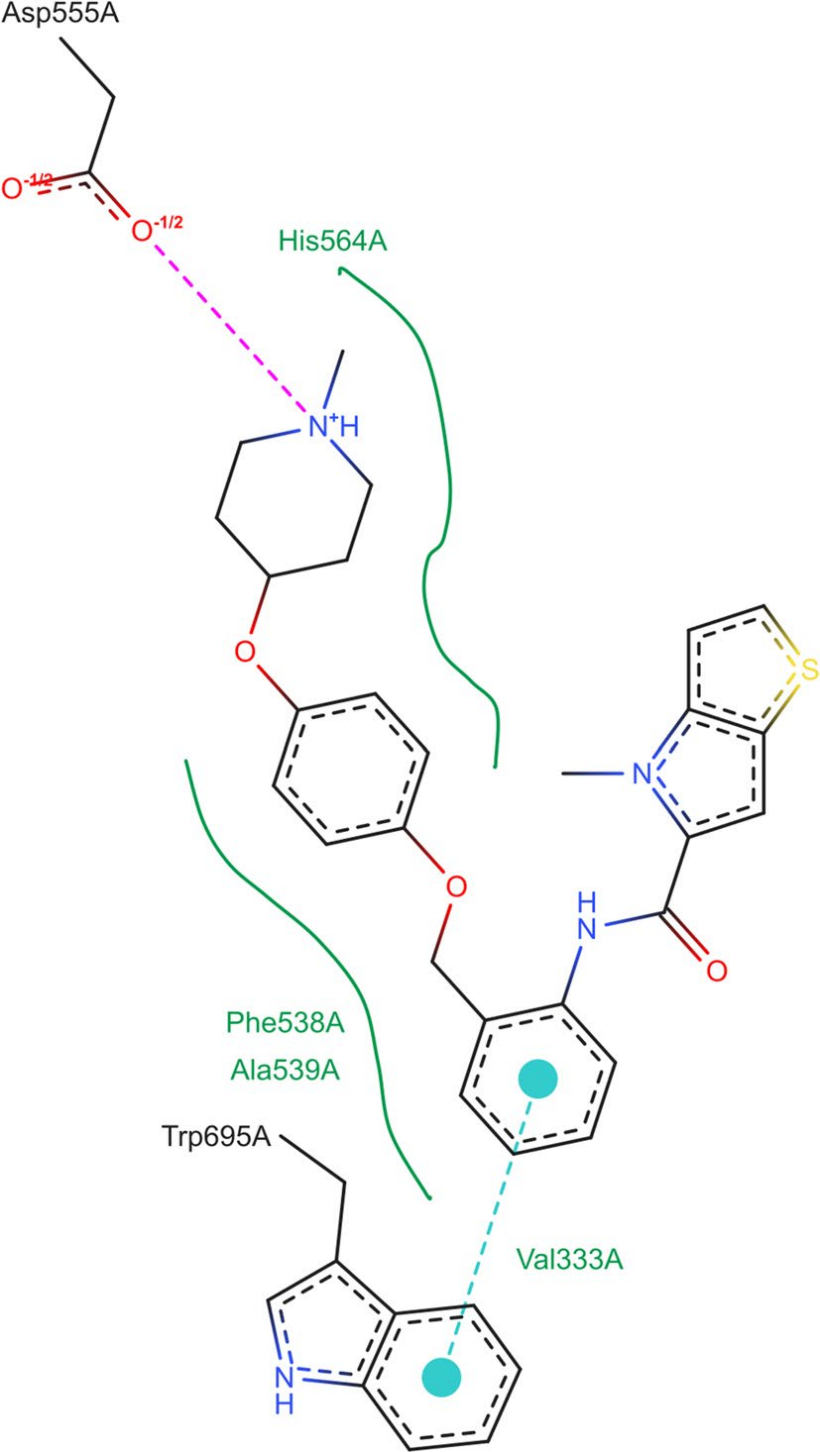
PoseEdit diagram of lysine-specific histone demethylase 1A in complex with an inhibitor 4-methyl-*N*-[2-[[4-(1-methylpiperidin-4-yl)oxyphenoxy]methyl]phenyl]thieno[3,2-b]pyrrole-5-carboxamide (PDB code: 5LGT; Proteins*Plus* identifier: 6W3_A_902). The following ligand interactions are depicted in the diagram: an ionic interaction with residue Asp555A, a pi–pi interaction with residue Trp695A, hydrophobic contacts with residues His564A, Phe538A, Ala539A, and Val333A

The affinity of a ligand does not depend on user’s taste. What may depend on user’s taste is the degree of focus to put on the various interactions contributing to ligand affinity. Interaction models are based on different studies and apply various different criteria to decide on the presence or absence of an interaction. The choice of the supported interaction types, their geometric parametrization, and the structure types as interaction partners may not always match the user’s expectations. Depending on the individual thresholds, experienced users might come to different assessments on the presence of specific interactions. Therefore, they might be skeptical that the automatically generated diagram accurately represents the chemical information they would have picked themself, or they might already be aware of discrepancies. This section will show how to explore the inhibitor’s environment beyond the pre-calculated diagram, providing the users with ideas to modify its chemical information content with PoseEdit. Figure 5 shows the PoseEdit diagram of the inhibitor 6W3_A_902 in complex with its target automatically generated by PoseView. The exported diagram in JSON format is available in the Supplementary Information (Online Resource 1).

Users can load and inspect the pose with the ligand-associated 3D binding site from the central *Pocket list* (Proteins*Plus* pocket identifier: FAD_A_901_6W3_A_902) in the 3D viewer. The PoseEdit diagram of the inhibitor is an excerpt from this 3D binding site. While the diagram displays ligand interactions with protein and nucleic acid residues and metals, the 3D binding site also shows all non-interacting structural elements and additional interaction partners that are not included in a PoseEdit diagram by default, such as water molecules. Therefore, the 3D binding site is a suitable starting point for verifying the chemical information content of the diagram resulting in ideas of how to extend it. The 2D-3D synchronization feature supports the user’s exploration of the ligand binding mode in both visualizations. Structural diagram objects in the 2D editor that are selected via the *Select mode* and consequently highlighted in dark green are automatically focused and highlighted in the 3D viewer as well. In addition, when users place the mouse pointer over any unselected or selected structural diagram object in the 2D editor or 3D viewer, it is highlighted with a light green color in both depictions. A *Select mode* option dictates how and what is selected. Users can select multiple atoms, bonds of structures, and structure circles via a rectangular and lasso selection tool with the *Rectangle* and *Lasso* option. With the *Click* option, users can pick single atoms, bonds, structure circles, as well as text labels.

Users can, for instance, select the atoms and bonds of all structures and the three text labels of the hydrophobic residues His564A, Phe538A, and Ala539A to highlight the corresponding structures in the 3D viewer. This feature enables an easier comprehension of already covered aspects of the 3D binding site and potential additional chemical information to be included. Interesting chemical information in the 3D binding site that is not depicted in the PoseEdit diagram is, for instance, the ligand FAD_A_901, the FAD cofactor. This cofactor interacts not only with the protein binding site via ionic interactions and hydrogen bonds but also with the inhibitor via pi-stacking interactions. Interactions with cofactors other than metal like FAD are not included in a PoseEdit diagram by default but might be relevant. The following section will provide more insights into the ligand binding mode of the cofactor by inspecting its PoseEdit diagram.

### Fixing of objective aesthetic deficiencies

**Fig. 6:**
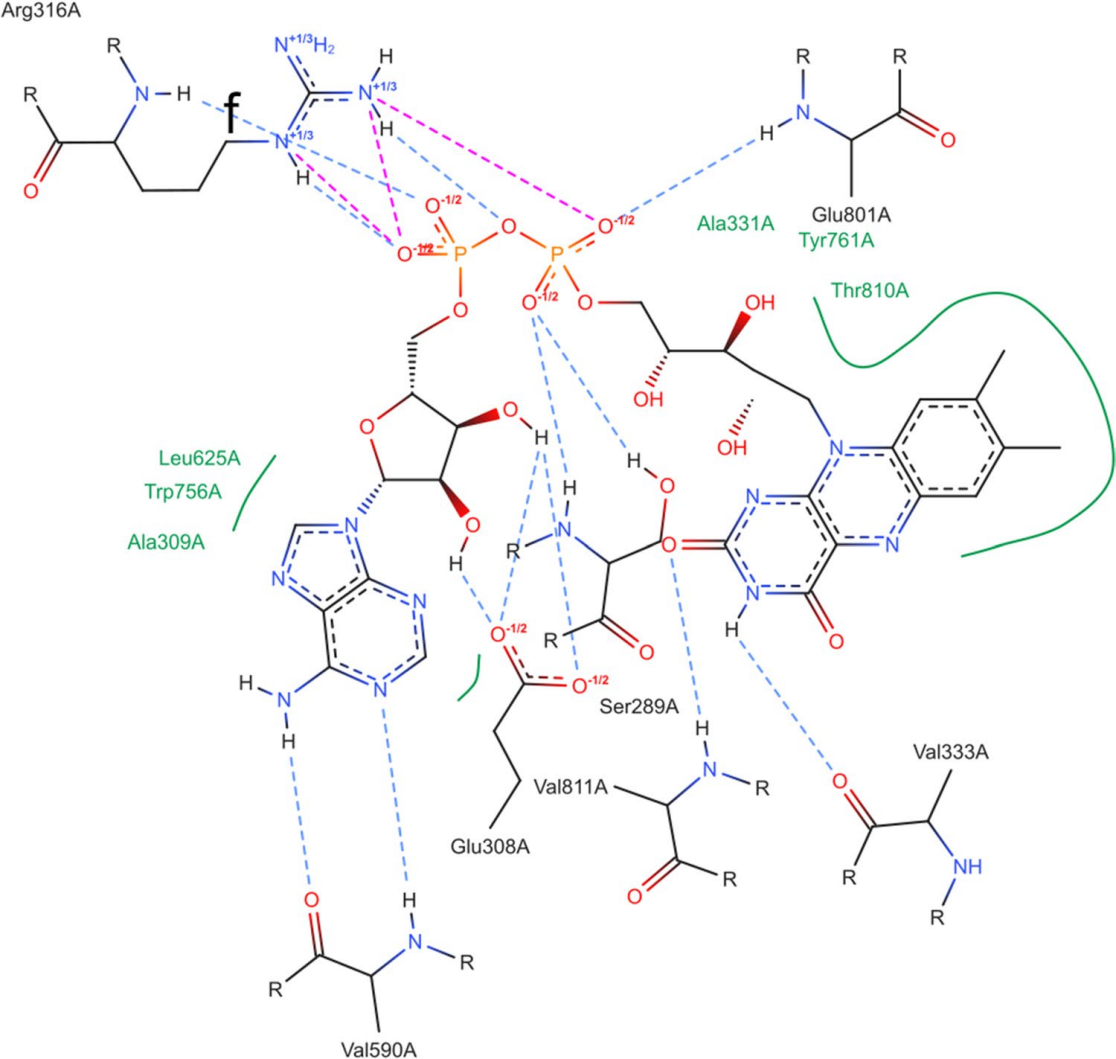
PoseEdit diagram of lysine-specific histone demethylase 1A in complex with a cofactor (PDB code: 5LGT; Proteins*Plus* identifier: FAD_A_901) with a suboptimal layout

**Fig. 7:**
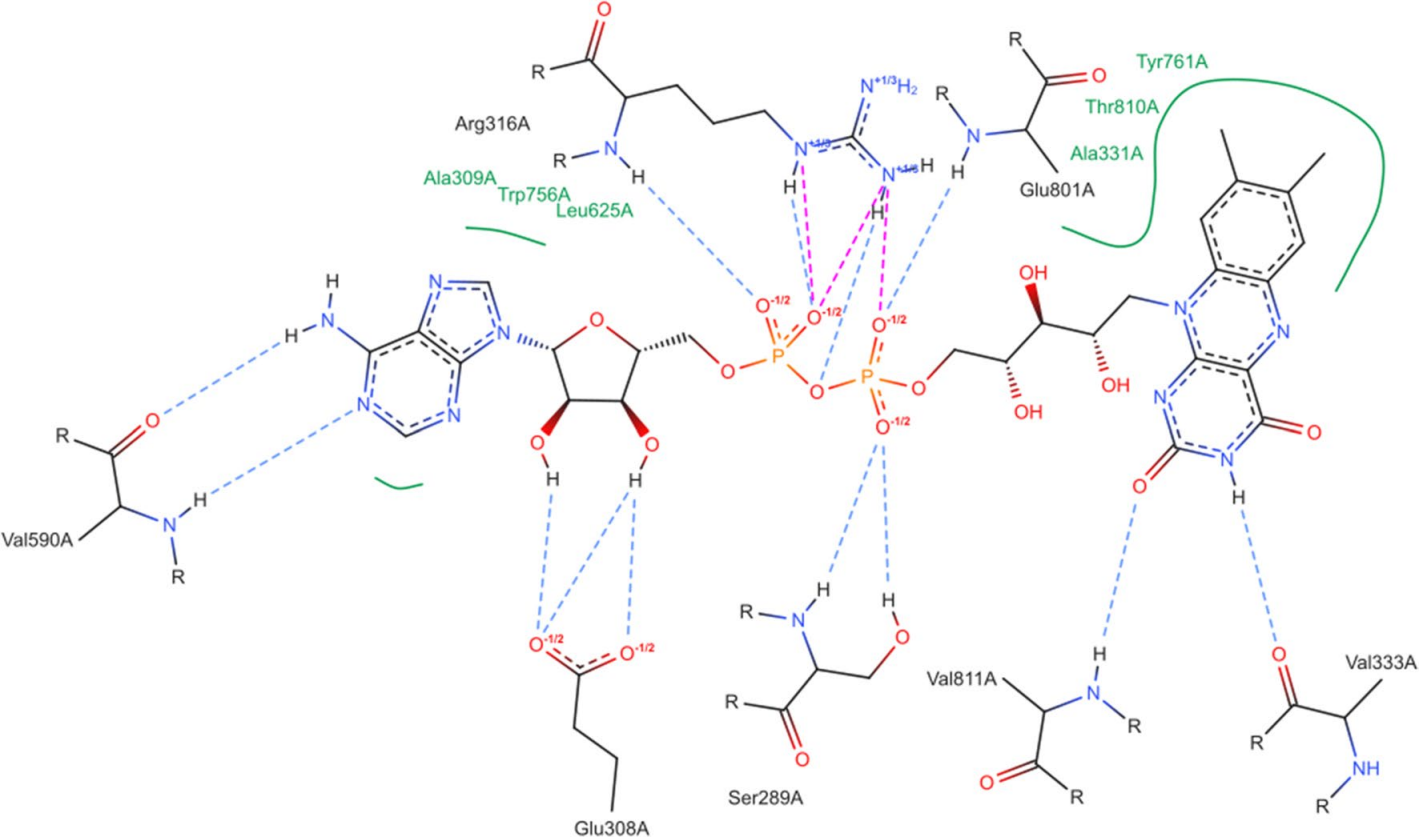
PoseEdit diagram of lysine-specific histone demethylase 1A in complex with a cofactor (PDB code: 5LGT; Proteins*Plus* identifier: FAD_A_901) with a layout optimized by PoseEdit

Concerning the occurrence of overlaps and intersections of graphical objects, especially those diagrams that condense a large amount of chemical information closely arranged in 3D space may need to be manually revised. A highly complex interaction pattern makes it algorithmically more challenging to depict a diagram in 2D, which may result in a lower layout quality. The diagram of the FAD cofactor mentioned previously is an example of such an objectively suboptimal layout caused, for example, by the adjacent and non-planar diphosphate group undergoing numerous hydrogen bond and ionic interactions. Such functional groups often contribute to crowded diagrams. Figure 6 shows the cofactor’s unmodified PoseEdit diagram. Figure 7 shows the diagram after manual optimization of the layout. The corresponding JSON files can be accessed in the Supplementary Information (Online Resource 2 and 3). A screen recording video that illustrates the following textually described diagram optimization procedure is given in Online Resource 4.

As the necessary modifications for optimizing a diagram may not be immediately visible, users might have to experiment with the editor’s functionality. The editor’s history, which is accessible via the *Undo* and *Redo* buttons enables, for instance, a trial-and-error approach. In addition, the possibility of hiding structures in the diagram via the editor’s top structure list helps to focus on a specific aesthetic issue and, consequently, quickly find ways to solve it.

The first aesthetic problem to fix is the curved ligand structure. This representation could be more appealing. In addition, the bent ligand structure surrounds and squashes the residues Glu308A, Val811A, and Ser289A such that the two hydrogen bonds of Glu308A cross the structure of Ser289A. The ligand structure can be elongated with the *Mirror mode* and the *Bond* option. By left-clicking on a bond, users can cycle through all possible mirroring positions until the most appropriate one is found. In this case, the *Mirror mode* is applied once on both phosphate anhydride bonds. Thereby, the diphosphate group is unchanged, and the non-bridging oxygen atoms that interact with Arg316A are still on the same side of the ligand, which prevents the crossing of Arg316A intersections with the ligand structure. Using the *Rotation mode*, the ligand is then rotated counterclockwise into a horizontal position. Subsequently, intersection- and overlap-free positions can now be found for all residues except Arg316A with the *Move mode* and *Rotation mode*. A structure’s mirroring, rotation, or movement also affects all associated interactions, hydrophobic contact splines, and text labels, simplifying such structural modifications. Further layout optimization can be achieved by reorienting the hydrogen atoms of the ligand towards the acceptor oxygen atoms of Glu308A by mirroring their bonds with the *Mirror mode* and by repositioning overlapping text labels of the hydrophobic contacts with the *Move mode*, which also creates more space for a better placement of Arg316A and Glu308A.

Next, we address the issue that the ligand’s diphosphate group engages in several intersecting hydrophilic interactions with Arg316A. No intersection-free position can be found for that residue by rotation and translation alone. By once mirroring the CB–CG bond of Arg316A with the *Mirror mode*, Arg316A can be moved and rotated such that its structure is not intersected anymore by the hydrogen bond that originates from its backbone. However, that hydrogen bond still intersects several interactions of the Arg316A side chain, which interact with a second non-bridging oxygen atom of the diphosphate group. Since the two non-bridging oxygen atoms are chemically equivalent in the diagram, users can avoid these intersections in two ways. Either users can remove the interactions of both non-bridging oxygen atoms with the *Remove mode* and add them with the *Add mode* to the equivalent one, or users can flip the positions of the two non-bridging oxygen atoms with the *Move mode*. The diagram is now free of overlaps and intersections and can be exported as a JSON file.

### Customization of the diagram

**Fig. 8:**
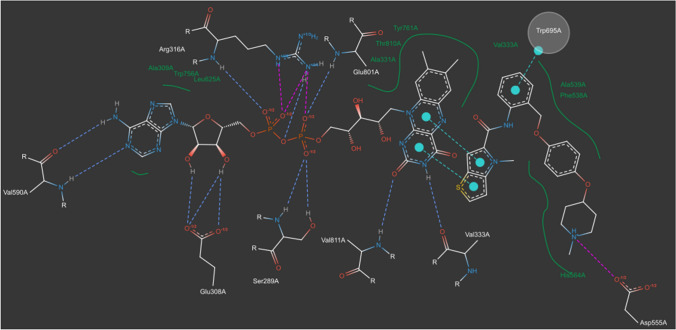
PoseEdit diagram obtained by the merging of the two diagrams of the LSD1 inhibitors with the ProteinsPlus identifier 6W3_A_902 and FAD and subsequential chemical editing and graphical restyling

This section will exemplify how to obtain a personalized diagram in terms of information content and graphical styles based on the diagram of the inhibitor and the optimized one of the FAD cofactor. Figure 8 shows an example of an individually customized diagram. The exported JSON file of the diagram is deposited in the Supplementary Information (Online Resource 5).

Users might, for instance, prefer a single, comprehensive diagram that includes the inhibitor, the cofactor, the interactions between the ligands, and their interactions with the protein binding site rather than two distinct diagrams. The information of the two diagrams can be combined by two approaches. The first one is to individually add the structures, interactions, hydrophobic contact splines, and text labels displayed in one diagram to another one with the *Add mode*. Structures can be specified by SMILES strings or via a list containing a preselection of frequently appearing binding site structures, from which users can select, for example, the interacting residues. Ligands like the inhibitor or FAD cofactor are not in the list and must be added via the corresponding SMILES strings, which can be obtained from the central *Ligand list*. Subsequently, the *Add mode* can be used to draw all missing interactions, hydrophobic contact splines, and text labels. The second and more straightforward and efficient approach is the merging of the two diagrams with the JSON import feature of PoseEdit. Users can, for example, export the JSON file of the diagram of the inhibitor and import it into the optimized diagram of FAD via the button with the JSON text label and plus sign. The imported diagram is automatically placed next to the one of FAD. Multiple structures can be selected and subsequently moved and rotated, along with all linked interactions, hydrophobic contact splines, and text labels using the *Select mode*’s rectangle or lasso selection tool. Based on the 3D binding site information, users can select all structures of the diagram of the inhibitor and apply the *Move mode* and *Rotation mode* such that the two interacting aromatic ring systems of both ligands are adjacently placed. The missing pi-stacking interactions between the ring systems can then be drawn with the *Add mode*.

Based on the merged diagram, another subjective adjustment exemplified here regards the Nε nitrogen atom of the Arg 316A side chain. This atom is involved in a hydrogen bond as well as an ionic interaction with the same ligand atom. Users might want to keep only the stronger intermolecular force, the ionic interaction. With the *Remove mode*, users can remove the nitrogen atom’s hydrogen bond and its explicitly drawn hydrogen atom. The nitrogen atom can then be annotated with one implicit hydrogen atom using the *Edit mode*.

Since the diagram is very complex, users might consider also reducing the diagram’s complexity to avoid overloading the viewer with information or to focus on specific aspects like the atomic interactions with the protein residues. In this regard, the *Edit mode* can be useful, for example by changing the representation style of Trp695A, which is involved in a pi-stacking interaction, to the *Circle representation*.

Finally, users can modify all graphical styles in the diagram via a comprehensive configuration list to further individualize the diagram. The list is accessible via the *Opts* button and contains numerous styling options for atoms, bonds, interactions, structures, structure circles, the editor’s control elements and the diagram background. Users can freely experiment with custom settings since the editor’s editing history tracks all changes. PoseEdit also offers a list of several preset themes. To exemplify the various styling possibilities, the *Dark theme*, which might be an eye strain-reducing alternative for some users, was used to recolor the background and structures in the diagram. The customized and final diagram now contains the user-desired chemical information and graphical styles.

## Conclusion

In this work, we presented PoseEdit as a comprehensively extended and interactive version of the tool PoseView. This new development stands out from other published tools in several ways. User preferences and aesthetic ideals cannot always be fully satisfied by automatically generated diagrams. Users working with 2D ligand interaction diagrams, and in particular those who favor the ones calculated by PoseView will clearly benefit from the extended opportunities of PoseEdit, avoiding typical limitations of commonly used 2D interaction diagram generators. PoseEdit enables users to resolve all sorts of subjective and objective deficiencies that would otherwise impede the intended communication of the ligand binding mode or even prevent diagram usability. This key feature distinguishes PoseEdit from other tools that provide either static diagrams or diagrams with a much lower level of interactivity.

Furthermore, while previously existing tools are all desktop applications that are not all freely accessible, PoseEdit can be accessed without limitations on a web server. This makes PoseEdit’s distinctive features usable for everyone and everywhere on various devices without any installation issues. The interactive diagrams are embedded in a 2D editor with an intuitive interface design making it easily accessible to all scientists. Several additional features, like the editing history or the diagram export/import functionality, render it a user-friendly alternative to other tools.

We hope that PoseEdit will increase the usage and quality of 2D ligand interaction diagrams by combining its algorithmically generated output with the valuable input of the scientist on a web-based platform. While the main application of PoseEdit is the generation of pose diagrams, the code base can be used for other purposes. For example, we are currently developing a two-dimensional editor for complex interaction patterns, which should substantially simplify the use of GeoMine [14, 15], our database of macromolecule-ligand complex structures.

## Supplementary Information

Below is the link to the electronic supplementary material.

Supplementary file1 (JSON 26 KB)

Supplementary file2 (JSON 50 KB)

Supplementary file3 (JSON 50 KB)

Supplementary file4 (MP4 169836 KB)

Supplementary file5 (JSON 69 KB)

## Data Availability

All data generated or analysed during this study are included in this published article and its supplementary information files.

## Abbreviations

IUPAC: Union of Pure and Applied Chemistry
JSON: JavaScript Object Notation
TXT: Text Document
PDB: Protein Data Bank
REST API: Representational State Transfer Application Programming Interface
SMILES: Simplified Molecular Input Line Entry System
SVG: Scalable Vector Graphics
3D: Three-dimensional
2D: Two-dimensional

## Software availability

The PoseEdit application is available at https://proteins.plus. The code of the InteractionDrawer library used for PoseEdit is available at https://github.com/rareylab/InteractionDrawer.
